# The Scavenger Receptor MARCO Expressed by Tumor-Associated Macrophages Are Highly Associated With Poor Pancreatic Cancer Prognosis

**DOI:** 10.3389/fonc.2021.771488

**Published:** 2021-10-29

**Authors:** Bian Shi, Junfeng Chu, Tao Huang, Xiaoqian Wang, Qiujian Li, Qilong Gao, Qingxin Xia, Suxia Luo

**Affiliations:** ^1^ Department of Integrated Traditional Chinese and Western Medicine, Affiliated Cancer Hospital of Zhengzhou University, Zhengzhou, China; ^2^ Department of Oncology, Affiliated Cancer Hospital of Zhengzhou University, Zhengzhou, China; ^3^ Department of Hepatopancreatobiliary Surgery, Affiliated Cancer Hospital of Zhengzhou University, Zhengzhou, China; ^4^ Department of Pathology, Affiliated Cancer Hospital of Zhengzhou University, Zhengzhou, China

**Keywords:** MARCO, tumor-associated macrophage, pancreatic cancer, prognosis, CD163

## Abstract

Macrophage-targeting therapies have become attractive strategies for immunotherapy. Deficiency of MARCO significantly inhibits tumor progression and metastasis in murine models of pancreatic cancer. However, the role of MARCO in patients with pancreatic cancer remains unclear. In the present study, we analyzed tumor-associated macrophage (TAM)-related changes using the Cancer Genome Atlas database. We observed a significant enrichment of M2 macrophages in pancreatic cancer tissues. We found that several pro-tumor markers are increased in cancer tissues, including CD163, CD206, SIRPα, LILRB1, SIGLEC10, AXL, MERTK, and MARCO. Crucially, MARCO is highly or exclusively expressed in pancreatic cancer across many types of solid tumors, suggesting its significant role in pancreatic cancer. Next, we investigated the expression of MARCO in relation to the macrophage marker CD163 in a treatment-naïve pancreatic cancer cohort after surgery (n = 65). MARCO and CD163 were analyzed using immunohistochemistry. We observed increased expression of CD163 and MARCO in pancreatic cancer tissues compared with paracancerous tissues. Furthermore, we observed a large variation in CD163 and MARCO expression in pancreatic cancer tissues among cases, suggesting the heterogeneous expression of these two markers among patients. Correlation to clinical data indicated a strong trend toward worse survival for patients with high CD163 and MARCO macrophage infiltration. Moreover, high CD163 and MARCO expression negatively affected the disease-free survival and overall survival rates of patients with pancreatic cancer. Univariate and multivariate analysis revealed that CD163 and MARCO expression was an independent indicator of pancreatic cancer prognosis. In conclusion, high CD163 and MARCO expression in cancer tissues is a negative prognostic marker for pancreatic cancer after surgery. Furthermore, anti-MARCO may be a novel therapy that is worth studying in depth.

## Introduction

Pancreatic cancer remains a highly lethal malignancy and is expected to be the second leading cause of cancer death in the United States within the next 20 to 30 years (1). In the United States, the 5-year survival rate at diagnosis of pancreatic cancer is approximately 10%, and even after surgery it is only approximately 20% (1, 2). Despite improvements in diagnosis and treatment options as well as rapid advances in targeted therapies and immunotherapy, the prognosis of patients with pancreatic cancer remains poor (2). Therefore, an urgent need exists to explore new biomarkers to have a clinically meaningful impact in the screening of patients with high-risk pancreatic cancer.

The tumor microenvironment (TME) has long been of great interest in a wide range of research studies in the field (3–5). Immune cells are a major component of the tumor microenvironment (6). In recent years, as research has progressed, many immune escape mechanisms have been reported, including T cells, tumor-associated macrophages (TAMs), myeloid-derived suppression cells (MDSCs), and natural killer (NK) cells (7–11). Among these immune cells, TAMs constitute the vast majority in the tumor microenvironment, which indicates the possibility that macrophage-targeting therapies are novel and attractive strategies for cancer treatment (12). Crucially, TAMs drive tumor progression through multiple mechanisms, which include increased angiogenesis, immunosuppression, and resistance to therapy (13). However, macrophages can also exhibit a proinflammatory phenotype that kills tumor cells effectively *in vitro* and *in vivo*. Antibodies to CD47 or SIRPα, developed against the TAM immunosuppression-related signaling pathway CD47-SIRPα (also known as the “don’t eat me” signal), allow macrophages to regain the ability to phagocytose tumor cells and restore the activity of CD8^+^ cytotoxic T cells, thereby significantly reducing tumor size and inhibiting tumor metastasis (14). Other preclinical trials targeting the macrophage “don’t eat me” signal have also yielded impressive results through the promotion of tumor cell phagocytosis by macrophages, such as those involving the inhibition of the MHC-I-LILRB1 axis, inhibition of the CD24-SIGLEC-10 axis, and CAR-M cellular immunotherapy (15–17). Despite these exciting findings, the role of macrophages in pancreatic cancer still needs to be fully uncovered.

Recently, studies have demonstrated that the scavenger receptor MARCO is mainly expressed by macrophages, and that higher MARCO expression is associated with the poor prognosis of many types of cancers (18–20). Importantly, preclinical studies have demonstrated that an anti-MARCO antibody inhibits tumor growth and metastasis in 4T1 mammary carcinoma and B16 melanoma mouse models (21). However, the association of decreased MARCO expression by macrophages with tumor progression and poor prognosis in human hepatocellular carcinoma (HCC) was also observed (22). The aforementioned studies suggest that MARCO expression may play distinct roles in different tumor types. Furthermore, in a murine model of pancreatic cancer, *Neyen* et al. found that MARCO deficiency significantly inhibits tumor progression and metastasis (23). MARCO antibody alters macrophage polarization, enhancing NK cell activation and tumor killing, and the anti-tumor effect of MARCO antibody in combination with PD-1/L1 antibody is more potent in a mouse model of melanoma (24). However, the roles of MARCO-expressing macrophages in human pancreatic cancer remain unclear.

In the present study, we characterized the gene expression profiles from the Cancer Genome Atlas (TCGA) data set (https://portal.gdc.com). Immune cell type analysis indicated that M2 macrophages, monocytes, and uncharacterized cells are significantly different between normal and pancreatic cancer tissues, which suggested that M2 macrophages may contribute more to the progression of pancreatic cancer. We then analyzed many correlated macrophage biomarkers and the results suggested that MARCO-expressing macrophages are highly associated with poor pancreatic cancer prognosis. Furthermore, MARCO expression was analyzed in many solid tumor types. Finally, we analyzed the expression profiles of CD163 and MARCO in both cancer and paracancerous tissues from patients with pancreatic cancer.

## Materials and Methods

### TCGA Data Analysis

#### (1) Gene Expression Data Sets

A gene expression data set of pancreatic cancer and normal tissues was acquired from the TCGA database (http://tcga-data.nci.nih.gov/tcga/tcgaAbout.jsp). The data were from 178 pancreatic cancer tissues, 4 paracancerous tissues, and 328 healthy tissues.

#### (2) Immune Infiltration Estimations

To produce reliable immune infiltration estimations, we used immunedeconv, an R package that integrates six state-of-the-art algorithms (quanTIseq). This R package and all of the aforementioned analysis methods were implemented in R Foundation for Statistical Computing (2020) version 4.0.3 and the software packages ggplot2 and pheatmap as previous described (25).

#### (3) Analysis of Differential Expression

The limma package (version: 3.40.2) for R was used to study the differential expression of mRNAs. The adjusted *P* value was analyzed to correct for false positive results in the TCGA or GTEx data set. The defined thresholds for screening for the differential expression of mRNAs were as follows: adjusted *P* < 0.05 and log (fold change) >1 or log (fold change) < −1. To further confirm the underlying function of potential targets, the data were analyzed using functional enrichment. Kyoto Encyclopedia of Genes and Genomes (KEGG) enrichment analysis is a practical resource for analytically studying gene functions and associated high-level genome functional information. To better understand the carcinogenesis of mRNAs, the ClusterProfiler package (version: 3.18.0) in R was employed to analyze and enrich the KEGG pathway as previous described (26).

#### (4) Kaplan–Meier Survival Analysis

For Kaplan–Meier curves, *P* values and hazard ratios with 95% confidence intervals were generated using log-rank tests and univariate Cox proportional hazards regression. All of the abovementioned analytical methods and R packages were performed using R software version v4.0.3 (R Foundation for Statistical Computing, 2020) as previous described (27); *P* < 0.05 was considered statistically significant.

### Cancer and Paracancerous Tissues From Pancreatic Cancers

Sixty-five patients with pancreatic cancer surgically treated at the Affiliated Cancer Hospital of Zhengzhou University between July 1, 2016 and June 1, 2018 were collected for this study. This study was approved by the Ethics Committee at the Affiliated Cancer Hospital of Zhengzhou University. Clinical parameters were obtained from the records of patients in the same hospital. All methods and procedures associated with this study were conducted in accordance with the Good Clinical Practice guidelines and accorded ethically with the principles of the Declaration of Helsinki and local laws.

### Immunohistochemistry and Image Analysis

Formalin-fixed and paraffin-embedded sections of pancreatic cancer tissue and paracancerous tissue (3–5 μm thick) were dewaxed and rehydrated. Antigen retrieval was performed by heating the slides in 10 mM Tris buffer with 1 mM EDTA (pH 9) in a streamer for 20 min. Then, endogenous peroxidase activity was inhibited through immersion in 3% H_2_O_2_ for 5 min. After washing with Tris-buffered saline containing Tween, endogenous biotin was inhibited through sequential incubation with 0.1% antibiotin protein and 0.01% biotin (Dako, Glostrup, Denmark), respectively, for 10 min at room temperature. Other nonspecific binding sites were blocked with 3% skimmed milk powder for 30 min at room temperature. Sections of pancreatic cancer tissue and paracancerous tissue were incubated with the monoclonal mouse antibody anti-human CD163(abcam, Cat#: ab182422) and MARCO (Bioss, Cat#: bs-2659R) for one night at 4°C. Subsequently, the sections were serially rinsed and incubated with secondary antibodies. Immunohistochemical staining was evaluated independently by two experienced pathologists blinded to the patients’ clinical characteristics and outcomes. The median was selected as the cutoff value for high or low CD163 and MARCO expression.

### Follow-Up and Survival Analysis

Disease-free survival (DFS) was calculated from the date of surgery to the time of recurrence or metastasis, and patients alive in a stable state were censored at the time of last contact (28). The overall survival (OS) was calculated from the date of surgery to the time of death, and patients who were alive at the time of last contact were censored (28). The DFS and OS were calculated using the Kaplan–Meier method. After surgery, all of the patients were scheduled for follow-up evaluations at our hospital from the date of initial treatment to the follow-up deadline of September 1, 2021 or to the time of death. Clinical examinations were performed by our oncology specialists every 3 months, including complete blood examinations and chest and abdominal computed tomography scans during the first 2-year period. From years 2 to 5, patients were examined every 6 months. Beyond 5 years, patients were examined every year. If follow-up evaluations revealed metastatic disease and/or local recurrence, other therapies were applied, including conventional therapies (surgery, chemotherapy, and radiotherapy) and immunotherapy.

### Statistical Analysis

The GraphPad Prism 9.0 and SPSS 24.0 software packages were used to perform the statistical analyses. The DFS and OS were calculated using the Kaplan–Meier method from the time of surgery. The prognostic factors were analyzed using univariate and multivariable Cox proportional hazards regression models. The χ^2^ test was used in a prespecified analysis to compare the characteristics of groups. Other data were analyzed using a *t* test. For all statistical analyses, significance was indicated at a level of *P* < 0.05.

## Results

### M2 Macrophages Are Significantly Heterogeneous Among Pancreatic Cancer Patients

**Figure 1:**
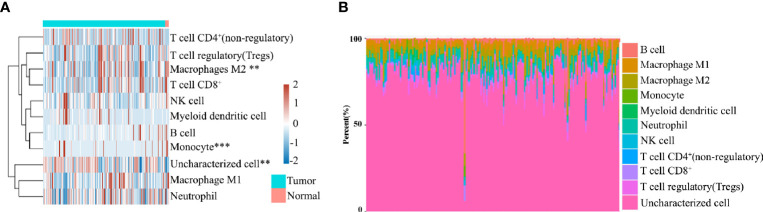
**(A)** Immune cell score heat map, where different colors represent the expression trend in different samples. **P < 0.01, ***P < 0.001. **(B)** The percentage abundance of tumor-infiltrating immune cells in each sample, with different colors and different types of immune cells.

To evaluate the significant changes of immune cells in patients with pancreatic cancer, RNA-seq data from the TCGA database were analyzed. Immune infiltration estimations were analyzed using quanTIseq. **Figures 1A, B** indicate that among the immune cells, M2 macrophages, monocytes, and uncharacterized cells in cancer tissues were significantly different compared with normal tissues. Furthermore, among tumors, the M2 macrophage content in each sample was also distinct, highlighting the heterogeneity in patients with pancreatic cancer patients (**Figures 1A, B**).

### Identification of Differentially Expressed Genes and Signal Pathways

**Figure 2:**
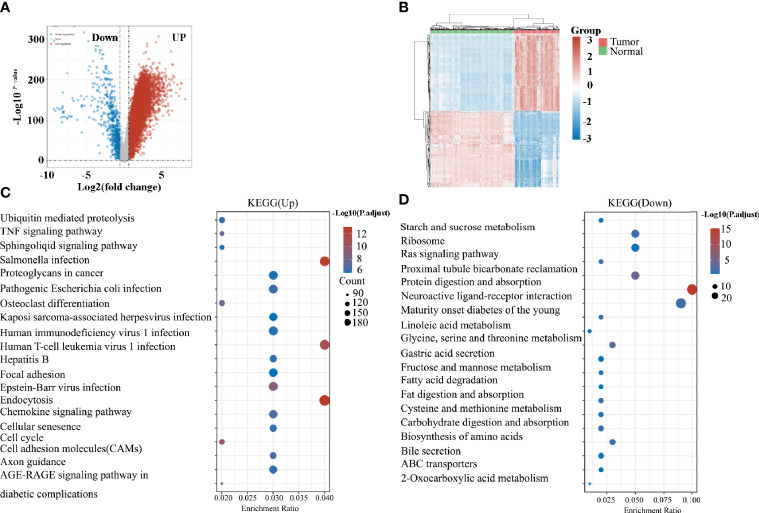
**(A)** Volcano plots constructed using fold-change values and adjusted *P* values. The red point in the plot represents the over-expressed mRNAs and the blue point indicates the down-expressed mRNAs with statistical significance. **(B)** Hierarchical clustering analysis of mRNAs, which were differentially expressed between tumor and normal tissues. **(C)** The top 20 upregulated KEGG signaling pathways in tumors. **(D)** The top 20 downregulated KEGG signaling pathways in tumors.

Next, we retrieved the transcriptome profiling data of pancreatic cancers from the TCGA database, which comprised 178 pancreatic cancer tissues, and 4 paracancer tissues. A total of 12 039 genes were distinguished as differentially expressed mRNAs, with 11 392 genes being upregulated and 647 genes being downregulated **(** **Figures 2A, B** **)**. The enriched KEGG signaling pathways were selected to demonstrate the primary biological actions of major potential mRNAs. The upregulated pathways related to TAMs included the osteoclast differentiation, endocytosis, the chemokine signaling pathway, and cell adhesion molecules (CAMs; **Figure 2C** **)**. By contrast, no highly relevant downregulated pathways were associated with TAMs **(** **Figure 2D** **)**. Taken together, the function of macrophages may play significant roles in pancreatic cancer. Thus, further studies on the roles of TAMs in pancreatic cancer are required.

### Several TAM-Related Molecules Are Significantly Increased in Patients With Pancreatic Cancer

**Figure 3:**
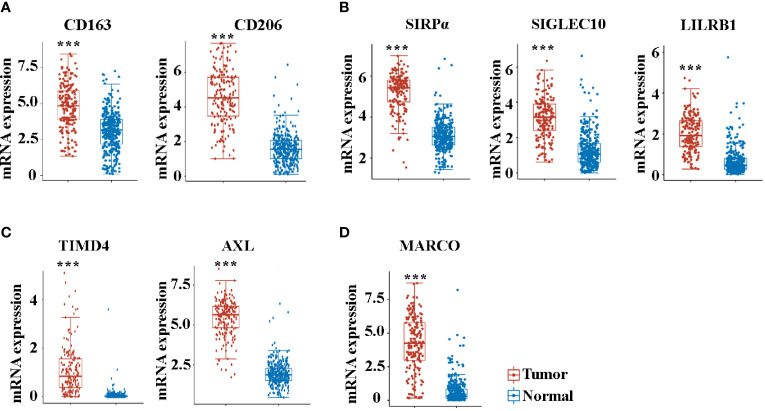
The mRNA expression of CD163 **(A)**, CD206 **(A)**, SIRPα **(B)**, SIGLEC10 **(B)**, LILRB1 **(B)**, TIMD4 **(C)**, AXL **(C)**, and MARCO **(D)** between cancer tissues and paracancer tissues in pancreatic cancer patients from the TCGA database. ***P < 0.001.

We then analyzed the expression of molecules that play a crucial role in the function of TAMs. Consistent with the increased enrichment of M2 macrophages, the M2 markers of CD163 and CD206 were significantly increased in pancreatic cancer compared with controls **(** **Figure 3A** **)**. Macrophage-mediated phagocytosis was inhibited by numerous “don’t eat me” signaling pathways, which mainly included CD47/SIRPα, CD24/SIGLEC10, and MHC-I/LILRB1 (15, 17, 29). Noteworthily, the expression of SIRPα, SIGLEC10, and LILRB1 also increased in cancer tissues **(** **Figure 3B** **)**. The AXL receptor tyrosine kinase (RTK) was implicated in the proliferation and invasion of many cancers, particularly in pancreatic ductal adenocarcinoma (PDAC) (30). Consistent with previous studies (30, 31), AXL and TIMD4 increased in patients with pancreatic cancer (**Figure 3C**). MARCO is a pattern recognition receptor that belongs to the class A scavenger receptor family (32). **Figure 3D** reveals that MARCO expression in pancreatic cancer is significantly increased.

### Prognostic Roles of TAM-Related Molecules

**Figure 4:**
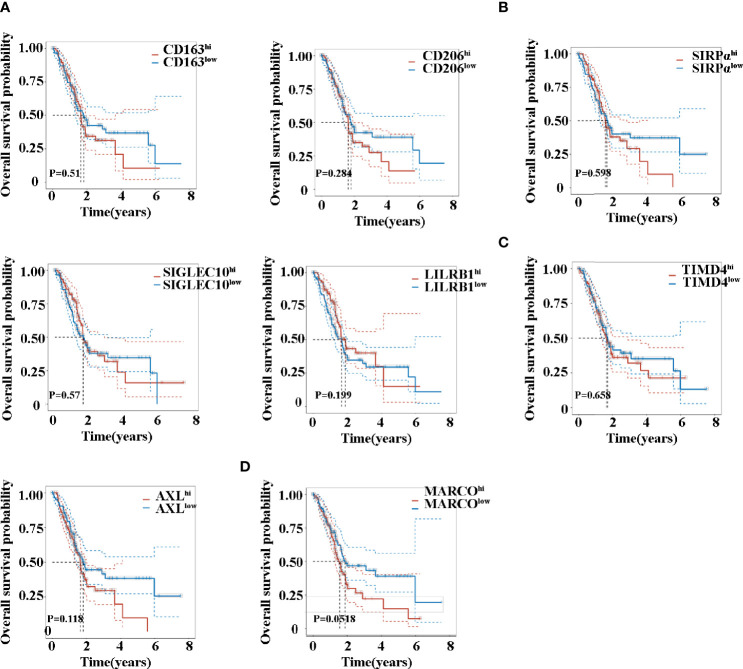
Overall survival time of patients with pancreatic cancer between high and low expression of CD163 **(A)**, CD206 **(A)**, SIRPα **(B)**, SIGLEC10 **(B)**, LILRB1 **(B)**, TIMD4 **(C)**, AXL **(C)**, and MARCO **(D)**.

Kaplan–Maier survival analysis with log-rank tests was also used to compare the difference in survival between high and low expression of TAM-related molecules. Noteworthily, the analysis revealed that patients exhibited a similar survival time based on the expression levels of CD163, CD206, SIRPα, SIGLEC10, LILRB1, and TIMD4 (**Figures 4A–C**). Previous studies have firmly demonstrated that the activation of the AXL RTK is associated with poor outcomes in PDAC (30, 33). Consistent with this, AXL ^hi^ (**Figure 4C**) patients exhibited a trend in shorter OS (*P* = 0.118). Although MARCO ^hi^ (**Figure 4D**) patients revealed a trend in shorter OS, no significant difference existed (*P* = 0.0518).

### MARCO Is Highly or Exclusively Expressed in Pancreatic Cancer Across Many Types of Solid Tumors

**Figure 5:**
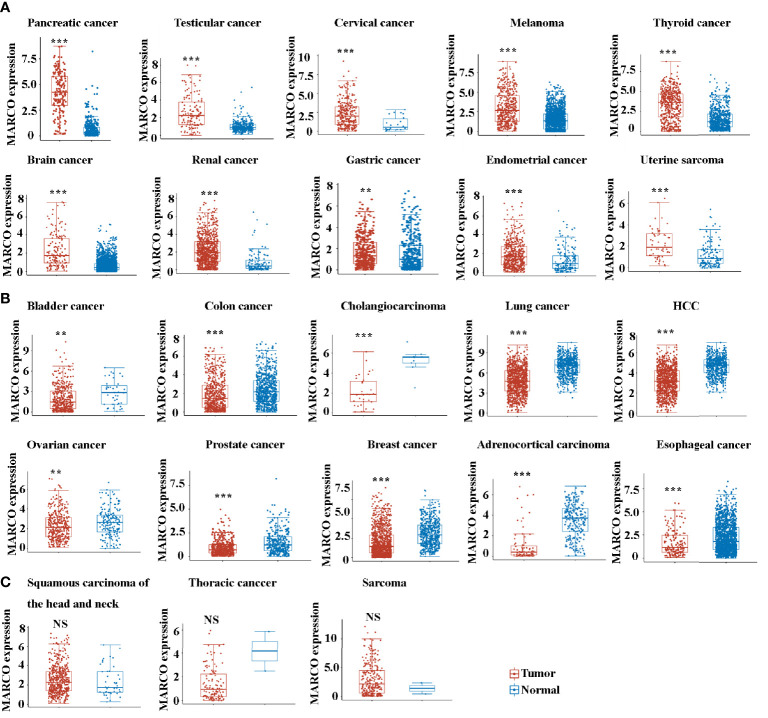
**(A)** The up-regulated mRNA expression of MARCO across many types of solid tumors from the TCGA database. **(B)** The down-regulated mRNA expression of MARCO across many types of solid tumors from the TCGA database. **(C)** The unchanged mRNA expression of MARCO across many types of solid tumors from the TCGA database. **P < 0.01, ***P < 0.001. *NS means no significant*.

Next, we compared the expression of MARCO between tumor tissues and control tissues across solid tumors. Noteworthily, a majority of solid tumors exhibited decreased MARCO expression compared with the controls, including bladder cancer, colon cancer, cholangiocarcinoma, lung cancer, HCC, ovarian cancer, prostate cancer, breast cancer, adrenocortical carcinoma, esophageal cancer, gastric cancer, endometrial cancer, and uterine sarcoma (**Figure 5B**). Although the expression of MARCO in testicular cancer, cervical cancer, melanoma, thyroid cancer, brain cancer, and renal cancer increased, there were fewer fold changes than in pancreatic cancer (**Figure 5A**). Additionally, the expression of MARCO in sarcoma, squamous carcinoma of the head and neck, and thoracic cancer did not change significantly (**Figures 5C**). Taken together, these data indicated that MARCO expression may play distinct roles in different cancer types. Furthermore, the highest expression of MARCO in pancreatic cancer suggested that MARCO may play a significant role in this type of cancer.

### MARCO Is Highly Expressed in Patients With Pancreatic Cancer

**Figure 6:**
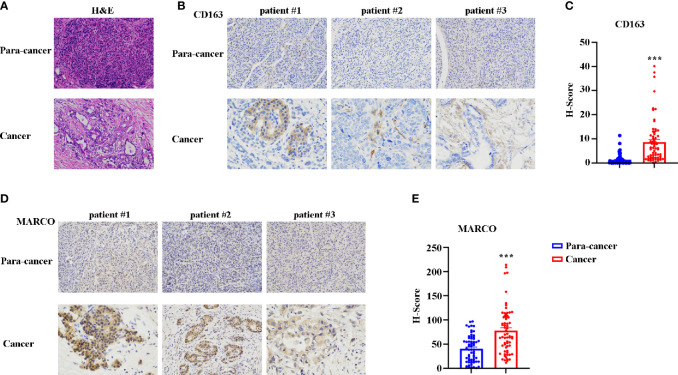
The expression of CD163 and MARCO in pancreatic cancer examined using immunohistochemistry (IHC). **(A)** Representative HE staining of cancer tissue and paracancer tissue in pancreatic cancer. **(B)** Representative IHC image of CD163 in pancreatic cancer tissues and paracancer tissues. **(C)** Quantitative analysis of CD163 expression between pancreatic cancer tissues and paracancer tissues. **(D)** Representative IHC image of MARCO in pancreatic cancer tissues and paracancer tissues. **(E)** Quantitative analysis of MARCO expression between pancreatic cancer tissues and paracancer tissues. ***P < 0.001.

Subsequently, we sought to assess the role of MARCO in pancreatic cancer using immunohistochemistry (IHC). In total, 65 cancerous tissues and paracancerous tissues were included. The tissues were taken from 43 male and 22 female patients with a median age of 63.5 years (range = 41-74 years). The characteristics of patients with pancreatic cancer from the Affiliated Cancer Hospital of Zhengzhou University are presented in **Table 1**. Before starting the immunohistochemical staining, we first confirmed the paracancerous and cancerous tissues using HE staining. **Figure 6A** presents representative HE images of paracancerous and cancerous tissues. Then, CD163 and MARCO expression in both paracancerous and cancerous tissues were examined using IHC. A comparison of CD163 expression between paracancerous and cancerous tissues revealed significantly higher expression in cancerous tissues than in paracancerous tissues **(** **Figures 6B, C** **)**. The expression of MARCO was also detected in paracancerous and cancerous tissues, and our data demonstrated that MARCO expression significantly increased in cancerous tissues compared with paracancerous tissues (**Figures 6D, E** **)**.

**Table 1:** Patient Characteristics.

Characteristic	No. of patients	%
**Gender**		
Male	43	66.15
Female	22	33.85
**Age(years)**		
Median	63.5	
Range	41-74	
**ECOG PS**		
0	53	81.54
1	12	18.46
**Site of primary tumor**		
head	14	21.54
body and tail	51	79.46
**Size of primary tumor(cm)**		
≥5	17	26.15
<5	48	73.85
**Histopathological grading**		
High	9	13.85
Intermediate	32	49.23
low	24	36.92
**TNM stage**		
I	33	50.77
II	20	30.77
III	12	18.46

### Relationship of MARCO With Clinicopathologic Features of Pancreatic Cancer Patients

CD163 and MARCO expression were diverse in each sample **(** **Figures 6C, E** **)**. We calculated the H-score of CD163 expression and divided the patients into high expression (CD163 ^hi^) and low expression (CD163 ^low^) subgroups according to a cutoff value of a mean H-score. We also classified the cohort into two subgroups according to the mean H-score of MARCO in tumor tissues. High CD163 expression was positively correlated with high ECOG PS (*P* = 0.002), high TNM stage (*P* = 0.000), and low histopathological grading (*P* = 0.006; **Table 2** **)**. Similarly, high MARCO expression in tumor tissues was positively correlated with high ECOG PS (*P* = 0.002), high TNM stage (*P* = 0.002), and low histopathological grading (*P* = 0.010; **Table 2** **)**.

**Table 2:** Correlation of clinicopathologic characteristics with CD163 and MARCO expression.

Characteristics	CD163			MARCO			
	High	Low	*P*-value	High	Low		*P*-value
**Age(years)**							
≤60	13	20		20		13	
>60	11	21	**0.675**	13		19	**0.107**
**Gender**							
Male	18	25		19		14	
Female	6	16	**0.249**	14		18	**0.265**
**ECOG PS**							
=0	15	38		22		31	
=1	9	3	**0.002**	11		1	**0.002**
**Site of primary tumor**							
Head	8	6		7		7	
Body and tail	16	35	**0.077**	26		25	**0.948**
**Size of primary tumor(cm)**							
≥5	9	8		10		7	
<5	15	33	**0.111**	23		25	**0.440**
**Histopathological grading**							
High	7	2		1		8	
Intermediate and low	17	39	**0.006**	32		24	**0.010**
**TNM stage**							
I and II	14	39		22		31	
III	10	2	**0.000**	11		1	**0.002**
**CA19-9 (U/mL)**							
<37	10	18		12		16	
≥37	14	23	**0.861**	21		16	**0.267**
**CEA (ng/mL**)							
<3.5	11	19		13		17	
≥3.5	13	22	**0.968**	20		15	**0.267**
**ALT(U/L)**							
<75	20	35		27		28	
≥75	4	6	**0.827**	6		4	**0.526**
**AST (U/L)**							
<75	19	34		28		25	
≥75	5	7	**0.706**	5		7	**0.485**
**Total bilirubin**							
<75	17	32		27		22	
≥75	7	9	**0.515**	6		10	**0.221**
**Direct bilirubin**							
<75	18	31		25		24	
≥75	6	10	**0.956**	8		8	**0.943**
**Indirect bilirubin**							
<75	18	30		24		24	
≥75	6	11	**0.871**	9		8	**0.835**
**WBC**							
<3.5x10^9^	5	9		6		8	
≥3.5x 10^9^	19	32	**0.916**	27		24	**0.504**
**RBC**							
<3.5x10^12^	4	6		5		5	
≥3.5x 10^12^	20	35	**0.827**	28		27	**0.958**
**HGB(g/dL)**							
<15	5	8		7		6	
≥15	19	33	**0.898**	26		26	**0.804**
**PLT**							
<100x10^9^	7	10		9		8	
≥100x 10^9^	17	31	**0.672**	24		24	**0.835**

### Prognostic Implication of MARCO in Patients With Pancreatic Cancer

**Figure 7:**
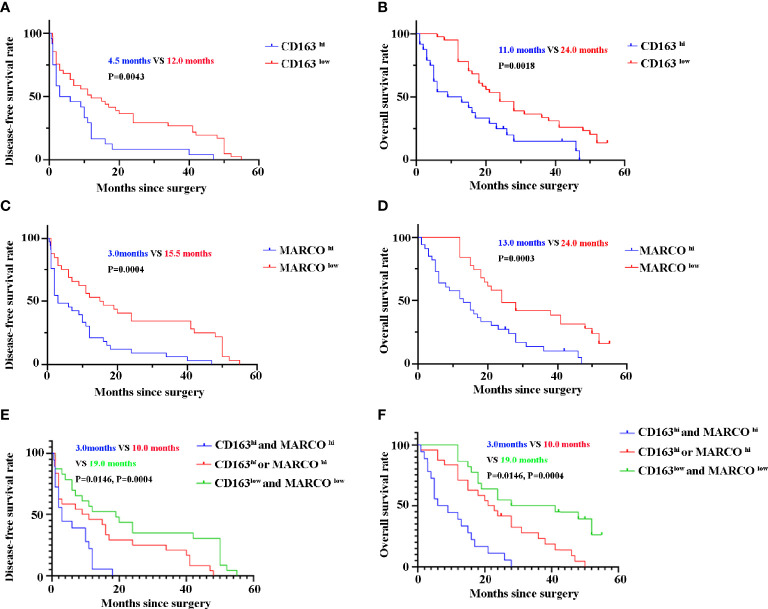
The disease-free survival (DFS) and overall survival (OS) of pancreatic cancer patients between high and low expression of CD163 and MARCO. **(A)** DFS curve of patients with pancreatic cancer between CD163 ^hi^ and CD163 ^low^. **(B)** OS curve of patients with pancreatic cancer between CD163 ^hi^ and CD163 ^low^. **(C)** DFS curve of patients with pancreatic cancer between MARCO ^hi^ and MARCO ^low^. **(D)** OS curve of patients with pancreatic cancer between MARCO ^hi^ and MARCO ^low^. **(E)** DFS curve of patients with pancreatic cancer between CD163^hi^ MARCO ^hi^ and others. **(F)** OS curve of patients with pancreatic cancer between CD163^hi^ MARCO ^hi^ and others.

Until the last follow-up, 55 patients died. CD163 ^hi^ patients with pancreatic cancer had a shorter DFS (4.5 months vs 12.0 months, *P* = 0.0043) and OS (11.0 months vs 24.0 months, *P* = 0.0018) than 163 ^low^ patients **(** **Figures 7A, B** **)**. Similarly, patients with pancreatic cancer with high MARCO expression in tumor tissues had a shorter DFS (3.0 months vs 15.5 months, *P* = 0.0004) and OS (13.0 months vs 24.0 months, *P* = 0.0003) than MARCO ^low^ patients **(** **Figures 7C, D** **)**. Furthermore, we analyzed the combined role of CD163 and MARCO in pancreatic cancer. Patients with pancreatic cancer with high expression of both CD163 and MARCO had the shortest DFS (3.0 months vs 10.0 months vs 19.0 months, *P* = 0.0146 and *P* = 0.0004, receptively) and OS (7.5 months vs 22.0 months vs 34.5 months, *P* = 0.0004 and *P* = 0.0004, respectively) among patients with high expression of only CD163 or MARCO and patients with low expression of both CD163 and MARCO (**Figures 7E, F** **)**. A univariate analysis indicated that high ECOG PS, larger tumor size, high TNM stage, and low histopathological grading were risk factors for both DFS and OS **(** **Table 3** **)**. Noteworthily, CD163 and MARCO expression as well as the combined expression of CD163 and MARCO in tumor tissues were also correlated with DFS and OS **(** **Table 3** **)**. These risk factors revealed by the univariate analysis were adopted as covariates in a multivariate Cox proportional hazards model. Noteworthily, high expression of CD163 and MARCO as well as their combined expression were still independent prognostic indicators for both DFS and OS **(** **Table 4** **)**.

**Table 3:** Univariate analysis.

Parameters	Hazard ratio	DFS 95% CI	*P*-value	Hazard ratio	OS 95% CI	*P*-value
Age, years (≤60 *VS* >60)	0.782	(0.589, 0.989)	**0.210**	0.887	(0.699-1.204)	**0.592**
Gender (male *VS* female)	1.056	(0.758, 1.389)	**0.635**	1.235	(0.913-1.508)	**0.312**
ECOG PS (0 *VS* 1)	1.932	(1.213-2.567)	**<0.001**	1.618	(1.264-2.108)	**0.005**
Site of primary tumor (head *VS* body and tail)	0.59	(0.198, 1.347)	**0.287**	0.866	(0.594-1.096)	**0.461**
Size of primary tumor (cm) (≥5 *VS*. <5)	1.74	(1.37, 1.825)	**0.059**	1.155	(0.906-1.499)	**0.307**
Histopathological grading (high *VS* intermediate and low)	1.896	(1.389-2.620)	**<0.001**	1.727	(1.314-2.323)	**0.002**
TNM stage (I and II *VS* III)	2.285	(1.94-3.05)	**<0.001**	2.104	(1.488-2.564)	**0.001**
CD163 (high *VS* low)	1.718	(1.325-2.236)	**0.002**	1.522	(1.104-1.992)	**0.012**
MARCO (high *VS* low)	1.598	(1.119-2.207)	**0.003**	1.486	(1.079-1.913)	**0.015**
CD163/MARCO (double high *VS* others)	1.635	(1.056-2.314)	**0.0025**	1.408	(0.998-1.875)	**0.017**

**Table 4:** Multivariate analysis.

Parameters	Hazard ratio	DFS 95% CI	*P*-value	Hazard ratio	OS 95% CI	*P*-value
ECOG PS (0 *VS *1)	1.302	(0.966, 1.822)	**0.081**	1.401	(0.887-1.791)	**0.074**
Histopathological grading (high *VS* intermediate and low)	1.031	(0.645, 1.288)	**0.663**	1.121	(0.810-1.532)	**0.582**
TNM stage (I and II *VS* III)	1.847	(1.222-2.469)	**<0.001**	1.774	(1.229-2.311)	**0.001**
CD163 (high *VS* low)	1.718	(1.158, 2.613)	**0.008**	1.532	(1.055-2.123)	**0.007**
MARCO (high *VS* low)	1.612	(1.118, 2.246)	**0.001**	1.452	(1.039-1.997)	**0.016**
CD163/MARCO (double high *VS* others)	1.506	(1.023-2.157)	**0.002**	1.389	(0.997-1.869)	**0.018**

## Discussion

Research advances in PD-1/PD-L1 immune negative regulatory signaling pathways have driven tremendous advances in cancer immunotherapy. However, clinical studies have demonstrated that only 25-30% of tumors suppress the immune response through the PD-1/PD-L1 pathway, whereas others escape the immune response through a different molecular pathway or mechanism (34). Therefore, the inhibition of the PD-1/PD-L1 pathway alone may not be sufficient. The search for mechanisms or immunosuppressive pathways other than PD-1/PD-L1 is of great clinical value. TAMs control the tumor microenvironment and shape anti-tumor responses, affecting the clinical response rate in many cancers, including pancreatic cancer (12, 35–37). In the present study, we analyzed pancreatic cancer data from previous TCGA bulk RNA-seq data and determined that M2 macrophages are significantly enriched in tumor tissues. The significantly increased M2 markers of CD163 and CD206 also confirmed this conclusion. The upregulated KEGG pathways in tumor tissues include the osteoclast differentiation, endocytosis, the chemokine signaling pathway, and CAMs, which are correlated to TAMs (38–43). Significantly, these pathways are correlated to tumor progression (38–43). However, the survival analysis indicated that no significant differences exist based on the expression levels of both CD163 and CD206. It is worth emphasizing that this analysis was based on the mRNA levels of CD163 and CD206; mRNA expression does not always represent protein expression. In previous studies that have used IHC or IF, CD163 ^hi^ or CD206 ^hi^ has been significantly correlated with shorter survival (44, 45). In the present study, we also confirmed the protein expression of CD163 in patients with pancreatic cancer using IHC, which exhibited increased expression in tumor tissues. Furthermore, patients with high CD163 expression exhibited shorter DFS and OS. The univariate and multivariate analyses also revealed that high CD163 expression is an independent prognostic marker.

Many studies have supported TAMs as being M2-like macrophages; however, experimental evidence suggests that TAMs are not only a unique and distinct M2 myeloid population but also that they share M1 and M2 signature polarization (46, 47). “Don’t eat me” signaling pathways, such as CD47-SIRPα, the MHC-I-LILRB1 axis, and the CD24-SIGLEC-10 axis can be expressed by all types of macrophages (48) and play crucial roles in inhibiting macrophage phagocytosis of tumor cells in numerous cancer types (13, 15, 17, 29). This study found SIRPα, LILRB1, and SIGLEC-10 to be increased in pancreatic cancer tissues. However, patients exhibited a similar survival time based on the expression levels of SIRPα, SIGLEC10, and LILRB1. Further studies are required to identify the roles of these markers in patients with pancreatic cancer. In general, AXL and TIMD4 function as two phagocytosis-related molecules in normal homeostasis (49, 50). Recently, AXL and TIMD4 have also been reported to be positively correlated to the poor progression of many cancers (31, 51–53). The present study found the mRNA expression levels of AXL and TIMD4 to be increased. Furthermore, TIMD4 was not found to be an independent prognostic marker in the survival analysis. By contrast, high AXL expression has a tread to be an independent poor prognostic marker in pancreatic cancer. Previous studies have also confirmed that AXL is critical in the progression and metastasis of pancreatic cancer (30, 33). Inhibiting AXL has extended survival, reduced primary and metastatic burden, and enhanced sensitivity to gemcitabine in pancreatic mouse models (30, 33). These data reveal that high AXL expression is a negative prognostic marker in pancreatic cancer.

MARCO-expressing macrophages are present in the tumor microenvironment in human breast cancer, metastatic melanoma, periampullary adenocarcinoma of the intestine, and non-small-cell lung cancer (18, 19, 32, 54). In the majority of cancers, macrophage expression of MARCO is correlated with an immunosuppressive phenotype (32). By contrast, decreased MARCO expression by macrophages is correlated with tumor progression and poor prognosis in HCC (22). The present study found that a majority of solid tumors have decreased MARCO expression compared with controls, including bladder cancer, colon cancer, cholangiocarcinoma, lung cancer, HCC, ovarian cancer, prostate cancer, breast cancer, adrenocortical carcinoma, esophageal cancer, gastric cancer, endometrial cancer, and uterine sarcoma. Although the expression of MARCO in testicular cancer, cervical cancer, melanoma, thyroid cancer, brain cancer, and renal cancer increase, there are fewer fold changes than in pancreatic cancer. Additionally, the expression of MARCO in sarcoma, squamous carcinoma of the head and neck, and thoracic cancer does not change significantly. These data suggest that MARCO expression may play a more significant role in pancreatic cancer compared with other tumor types. Further studies are required to uncover the roles of MARCO in distinct tumor types.

Although MARCO deficiency significantly inhibited tumor progression and metastasis in a pancreatic mouse model (23), the role of MARCO in human pancreatic cancer has not been previously reported. In the present study, we first confirmed that MARCO expression increased in pancreatic cancer tissues compared with paracancerous control tissues using IHC. Critically, patients with pancreatic cancer with high MARCO expression exhibited shorter DFS and OS. The univariate and multivariate analyses also demonstrated that high MARCO expression is an independent prognostic marker in pancreatic cancer. Furthermore, high expression of both CD163 and MARCO was associated with the shortest DFS and OS. The univariate and multivariate analyses confirmed that combined CD163 and MARCO expression is an independent prognostic marker in pancreatic cancer. Therefore, we first determined that MARCO expression is a biomarker that predicts the progression of pancreatic cancer.

Our study has several limitations that require consideration. Although our IHC approach revealed that CD163 and MARCO expression in pancreatic cancer significantly increases and high CD163 and MARCO expression is correlated with poor prognosis, a more detailed understanding of MARCO^+^ macrophages is required, which can be obtained using more experimental methods such as Sc-RNA-seq, RNA-seq, and multiplex immunofluorescence. Nevertheless, the expression of CD163 and MARCO is still a good predictor of pancreatic cancer prognosis. Furthermore, anti-MARCO therapy may be a novel approach that is worth studying in depth. Lastly, independent studies are required to confirm the findings of this study.

## Data Availability Statement

The original contributions presented in the study are included in the article/supplementary material. Further inquiries can be directed to the corresponding author.

## Ethics Statement

This study was reviewed and approved by the Ethics Committee at the Affiliated Cancer Hospital of Zhengzhou University. The patients/participants provided their written informed consent to participate in this study.

## Author Contributions

BS and JC performed the experiment, wrote the manuscript, and finished the figures and tables. TH, XW, QL, and QX read and edited the manuscript. SL designed experiments, read and edited the manuscript. All authors contributed to the article and approved the submitted version.

## Funding

This work was supported by Key Research Project of Henan Province (32211041).

## Conflict of Interest

The authors declare that the research was conducted in the absence of any commercial or financial relationships that could be construed as a potential conflict of interest.

## Publisher’s Note

All claims expressed in this article are solely those of the authors and do not necessarily represent those of their affiliated organizations, or those of the publisher, the editors and the reviewers. Any product that may be evaluated in this article, or claim that may be made by its manufacturer, is not guaranteed or endorsed by the publisher.
